# Single-cell transcriptome and epigenomic reprogramming of cardiomyocyte-derived cardiac progenitor cells

**DOI:** 10.1038/sdata.2016.79

**Published:** 2016-09-13

**Authors:** Xin Chen, Tushar Chakravarty, Yiqiang Zhang, Xiaojin Li, Jiang F. Zhong, Charles Wang

**Affiliations:** 1 Center for Genomics & Department of Basic Sciences, School of Medicine, Loma Linda University, Loma Linda, California 92350, USA; 2 Division of Cardiology, Department of Medicine, and Center for Cardiovascular Biology, and Institute for Stem Cell and Regenerative Medicine, University of Washington, Seattle, Washington 98109, USA; 3 CardioDx, Inc., 600 Saginaw Drive, Redwood City, California 94063, USA; 4 Division of Periodontology, Diagnostic Sciences & Dental Hygiene & Biomedical Sciences, Herman Ostrow School of Dentistry, and Norris Cancer Center, University of Southern California, Los Angeles, Los Angeles, California 90089, USA; 5 These authors contributed equally to this work

**Keywords:** Epigenomics, DNA methylation, Differentiation, Microarray analysis, Cardiac regeneration

## Abstract

The molecular basis underlying the dedifferentiation of mammalian adult cardiomyocytes (ACMs) into myocyte-derived cardiac progenitor cells (mCPCs) during cardiac tissue regeneration is poorly understood. We present data integrating single-cell transcriptome and whole-genome DNA methylome analyses of mouse mCPCs to understand the epigenomic reprogramming governing their intrinsic cellular plasticity. Compared to parental cardiomyocytes, mCPCs display epigenomic reprogramming with many differentially-methylated regions, both hypermethylated and hypomethylated, across the entire genome. Correlating well with the methylome, our single-cell transcriptomic data show that the genes encoding cardiac structure and function proteins are remarkably down-regulated in mCPCs, while those for cell cycle, proliferation, and stemness are significantly up-regulated. In addition, implanting mCPCs into infarcted mouse myocardium improves cardiac function with augmented left ventricular ejection fraction. This dataset suggests that the cellular plasticity of mammalian cardiomyocytes is the result of a well-orchestrated epigenomic reprogramming and a subsequent global transcriptomic alteration. Understanding cardiomyocyte epigenomic reprogramming may enable the design of future clinical therapies that induce cardiac regeneration, and prevent heart failure.

## Background and Summary

Heart muscle cells in lower vertebrates such as zebrafish can be substantially regenerated by dedifferentiation and proliferation of pre-existing cardiomyocytes^1,2^. On the other hand, the adult mammalian heart has long been thought to be a non-regenerative organ. This dogma has been challenged by increasing evidence demonstrating that postnatal cardiomyocytes do proliferate at a low rate and contribute to myocardial renewal^3–5^. Using a genetic cell fate mapping system and a pure myocyte culture technique, we recently demonstrated that mature mammalian cardiomyocytes can spontaneously dedifferentiate, re-enter cell cycle, and regain properties of mCPCs when cultured under specific growth conditions for a prolonged period. Such dedifferentiated cells can re-differentiate into cardiomyocytes with spontaneous contractile activity^6,7^. However, the molecular mechanism regulating the spontaneous dedifferentiation of the adult cardiomyocytes into mCPCs is not yet understood. It is unknown if there is a genome-wide epigenomic reprograming, e.g., change of the methylome, which results in a transcriptomic alteration in mCPCs. The purpose of the data set presented herein is to test the hypothesis that genome-wide epigenomic reprogramming, e.g., change of DNA methylome, underlies the transcriptomic alteration and the spontaneous dedifferentiation of ACMs.

Although all cells in an individual organism or tissue may have a virtually identical genome, each cell has a unique transcriptome that reflects the expression of a subset of genes, which can be affected by epigenetic states. Single-cell transcriptome analysis allows us to access the gene regulatory network at a whole-genome scale to identify genes and pathways that underlie the given cell type’s physiological functions, behavior and phenotype during development^8^. Since dedifferentiation and cellular reprogramming are often asynchronous^9^, it is essential to investigate the transcriptome at a single-cell level to better elucidate these underlying molecular mechanisms. Moreover, cell-to-cell variations in gene expression are critical in the development of many tissues^10,11^. Although this variation is especially important for stem cell differentiation and cellular dedifferentiation, it has been extremely challenging to measure and interpret data from a single cell in terms of genome-wide transcriptional activity due to random biological variation, which in certain conditions may not be functionally consequential, in addition to inherent system measurement errors^12–14^.

Single-cell sequencing technologies have been advanced remarkably recently, which allow us to gain a greater insight into cellular and molecular heterogeneity in multilayers of gene regulation that ultimately governs cell functions and behaviors. Such studies provide better strategy in controlling the onset and progression of many cellular events, such as lineage specification, differentiation and growth, and cell cycle control, which were exemplified in developmental biology, cancer/tumor, and immune system^15–19^. Our study at the time of experiment back a few years ago was limited in analyzing DNA methylome and transcriptome in the same single-cells. However, now it is possible to perform parallel multi-omics study from the same single cell to integrate transcriptomics with epigenomics including DNA methylome (e.g., scM&T- seq), histone modifications, chromatin accessibility, and chromosome states, or with genetics (e.g., copy number variation performed in scTri-seq)^20–25^. It is also highly desirable to further overlay the proteome with the information from genomic, epigeomic and transcriptomic levels to fully understand the developmental and pathophysiological process at single-cell level. We believe that although cardiovascular diseases such as heart failure can be secondary to other stress and injuries, single-cell functional genomics in cardiomyocytes during development and disease remodeling provide promising targets for promote cardiomyocyte generation thereby cardiac myogenesis.

**Figure 1:**
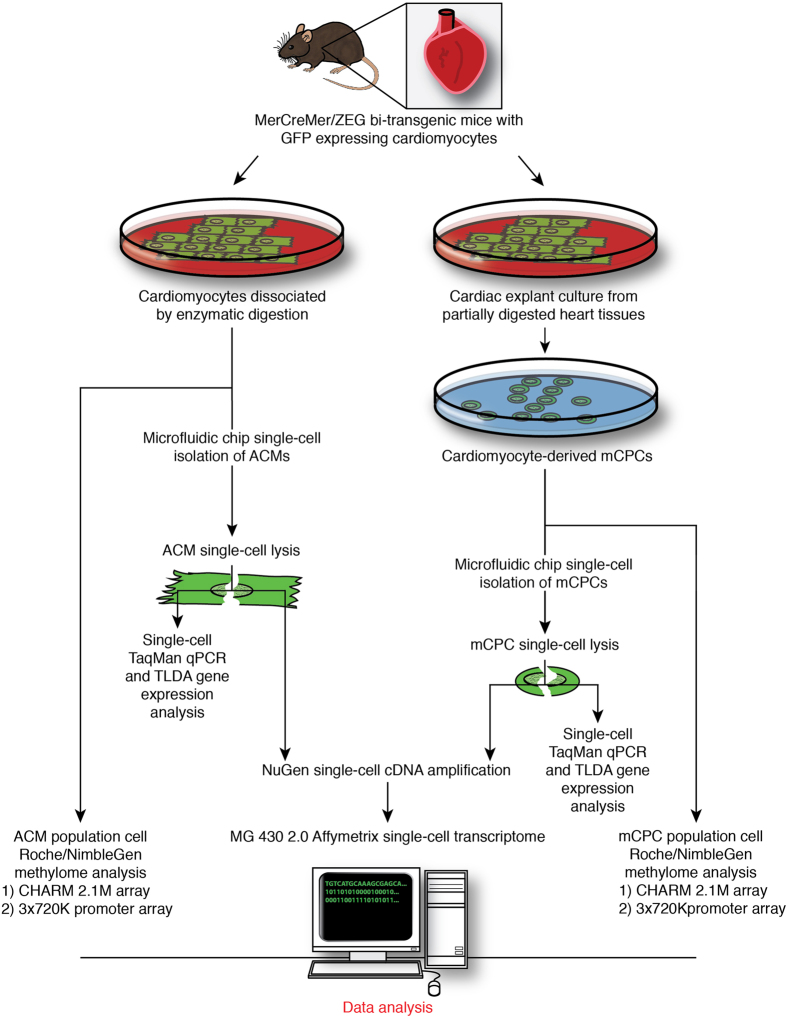
Flow chart of the experimental design and data analysis. The left and right arms depict the control and experimental groups’ workflow, respectively.

We present a whole-transcriptome analysis at single-cell level of freshly-isolated adult cardiomyocytes (controls) and mCPCs using a microfludic chip coupled with Affymetrix Mouse Genome 430 2.0 Array (Fig. 1) to understand the molecular mechanisms regulating adult cardiomyocyte dedifferentiation and reprogramming. Furthermore, using two different types of NimbleGen whole-genome tiling arrays including the Comprehensive High-throughput Arrays for Relative Methylation (CHARM) NimbleGen Mouse CHARM 2.1M Tiling Array (Feinberg_MM8_Me_HX1) and the NimbleGen Mouse DNA Methylation 3×720 K CpG Islands Pus RefSeq Promoter Tiling Array (100718_MM9_CpG_Refseq_Prom_MeDIP) for DNA methylome analyses (Fig. 1), we found that mCPCs displayed an epigenomic reprogramming as compared to ACMs, with many differentially-methylated regions, both hypermethylated and hypomethylated across the entire genome which was well correlated with the transcriptomic change. We observed an inverse correlation between global DNA methylation and transcriptomic expression, i.e., at methylome level, many genes were hyper-methylated in the promoter regions with a corresponding down-regulation of their transcripts in mCPCs (main paper published in Nature’s *Sci. Reports*)^7^. Therefore, our results demonstrate an orchestrated genome-wide epigenomic reprogramming and a subsequent transcriptomic change in mCPCs with a molecular signature displaying cell cycle reprogramming and acquired stemness. For simplicity, we will refer the two types of NimbleGen Mouse methylation arrays as NimbleGen Mouse CHARM 2.1M Array and NimbleGen Mouse 3×720 K Array, respectively, in the following sections.

## Methods

### Experiment study design

The overall experimental study design was illustrated in Fig. 1, which highlighted the major steps of the study from cardiomyocyte reprogramming, single cell isolations, single-cell whole transcriptome analysis, population cell DNA methylome analyses, and single-cell level gene expression validation.

### Bi-transgenic mouse model

All experimental protocols were approved by the Institutional Biosafety Committee and Animal Care and Use Committee at Cedars-Sinai Medical Center. The methods were carried out in accordance with the approved guidelines. Bi-transgenic MerCreMer/ZEG mice were produced by cross-breeding the cardiomyocyte-specific αMHC-MerCreMer mice and the ZEG reporter mice (Jackson Laboratory) as described previously^6,26–28^. To induce Cre-mediated gene recombination for GFP labeling specifically in cardiomyocytes, genotype-verified double heterozygous MerCreMer-Z/EG mice (2-month old) were treated with 4-OH-tamoxifen for 2 weeks followed by a waiting period of 2 weeks as described previously^6,9^. Cardiomyocytes were isolated using enzymatic dissociation method as described^6,9^.

### Adult cardiomyocyte enrichment

Adult mouse cardiomyocytes were enriched as previously described^6^. Briefly, ~3 month old bi-transgenic mouse hearts were enzymatically digested to dissociate ACMs. The non-cardiomyocyte cell types were removed from a total cell population by sequential sedimentation and Percoll gradient centrifugation. The enriched cardiomyocytes were then pre-plated in preparations of over 100,000 cells onto 22 mm culture glasses and assayed for positive and negative expression markers via confocal microscopy. RT-PCR amplification of cardiomyocyte expression markers was also conducted to confirm their enrichment.

### mCPC enrichment

To maximize the viability of transgenic mouse myocytes and follow their dedifferentiation, modified cardiac explant culture techniques described previously were used to generate myocyte-derived cardiac progenitor cells (mCPCs)^6,29^. Briefly, tamoxifen-treated bitransgenic mouse hearts were partially digested in calcium-free Tyrode’s physiological solution supplemented with 0.15 Wünsch unitml^−1^ of collagenase made from Liberase Blendzyme 4 (Roche Applied Science), followed by washing for 3 min in KB solution. The hearts were cut into small pieces in ~0.1 mm^3^ and rinsed in KB solution for 3 times. Tissues were transferred to laminin-coated 10 mm tissue culture dishes, with M199 medium containing 100 UmL^−1^ penicillin, 100 μgmL^−1^ streptomycin, 5% FBS (Invitrogen), 25 μM Blebbistatin, ITS (5 μgml^−1^ insulin and transferrin, 5 ngml^−1^ selenium), and 10 mM β-hydroxybutyric acid for the first two days of culture. Blebbistatin, ITS, and β-hydroxybutyric acid were replaced with bFGF 0.1 ngml^−1^ and TGF-β3 1 ngml^−1^, and FBS increased to 20% in subsequent cell culture. Medium was partially replaced every 2–3 days. Loosely adherent mCPCs were harvested by gentle pipetting 3 times with a disposable transfer pipette. Flow cytometry was used to characterize the immuno phenotypes of cardiac explant-derived cells as described previously^30^. Dedifferentiated cells spontaneously flattened, lost their striation and inward rectifier potassium current (decreased electrical capacitance), and ultimately re-entered the cell cycle as GFP-expressing mCPCs.

### Microfluidic capture of single cardiomyocytes and single cardiac progenitor cells

Freshly isolated adult (~3-month old) cardiomyocytes and mCPCs (derived from GFP-myocytes) were captured using microfludic chip (Fig. 1f in the accompanying paper)^31,32^. The adult cardiomyocytes were used as controls. Briefly, isolated adult cardiomyocytes were preserved in EGTA-free KB solution^6^, while mCPCs were kept in cold PBS during microfludic separation. Single-cells were isolated by encapsulation in droplet using a microfluidic device as described previously^32,33^. Once individual cells were isolated and verified under the microscope, each was lysed with 2 μl of a cell lysis buffer for cDNA amplification and single-cell whole transcriptome profiling. Alternatively, 5 μl Cell-to-Ct lysis buffer supplemented with DNase I (Ambion) was used to lyse each single cell for the validation of gene expression by Real-Time TaqMan single qPCR assays or by TLDA qPCR array.

### cDNA synthesis and amplification from single cardiomyocytes and single cardiac progenitor cells

Single-cell cDNA synthesis and amplification were performed using NuGen’s Ovation One-Direct System. Briefly, the first strand cDNA was synthesized from single cell lysate using a unique first strand DNA/RNA chimeric primer mix and reverse transcriptase. The primers had a DNA portion that hybridizes either to the 5′ portion of the poly (A) sequence or randomly across the transcript. Reverse-transcription extended the 3′ DNA end of each primer generating first strand cDNA. The resulting cDNA/mRNA hybrid molecule contained a unique RNA sequence at the 5′ end of the cDNA strand and subsequently, double stranded cDNA with DNA/RNA heteroduplex at one end was generated. Ribo-SPIA amplification of cDNA produced enough amount of cDNA for labeling and microarray hybridization.

### Single-cell whole-transcriptomic profiling by microarray

For each single cell, 5 μg of amplified cDNA was fragmented and used for biotin labeling with NuGen’s Encore Biotin Module. The biotin labeled cDNA was hybridized for 40 h with Affymetrix Mouse Genome 430 2.0 array. After the hybridization, the chips were washed with GeneChip Fluidics Station 450 (Affymetrix) according to the standard fluidic protocol EUKGE-WS2V5_450 (Affymetrix). Microarray images were acquired and processed using GeneChip Scanner 3000 7G (Affymetrix) and gene expression values were analyzed using Affymetrix Gene Expression Console^34,35^.

### DNA methylome analysis by microarrays

Genomic DNA was isolated from population adult cardiomyocytes and population mCPCs using Qiagen AllPrep DNA/RNA Micro Kit. Modified CHARM protocol with reduced starting amount of genomic DNA (500 ng), and amplified gDNA post-McrBC digestion (or without digestion) triplicated in each group, was used for two types of arrays^36,37^. Restriction enzyme McrBC was used to digest genomic DNA as it recognizes the site (A/G)^m^C(N_40–3000_)(A/G) ^m^C, with an optimal separation of 55–103 bp, covering nearly half of all possible 5-methylcytosine nucleotides in the genome^38^. For each sample, half of the fragmented gDNA was subjected to McrBC digestion thereby methylated cytosines were cut into smaller fragments; the other half was not treated with McrBC enzyme. Both McrBC-treated and untreated portions were fractionated by 1% agarose gel according to the CHARM protocol. The McrBC-treated portion was methyl-depleted (MD) DNA and the untreated (UT) portion represented the total genomic DNA input. Recovered MD and UT DNA from agarose gel were subsequently amplified using a GenomePlex Complete Whole Genome Amplification (WGA2) kit (Sigma) according to manufacture protocol. Amplified MD (equivalent to experimental sample) and UT (equivalent to input/control sample) samples were labeled with Cy5 and Cy3, respectively, according to standard NimbleGen Array protocol. Relative DNA Methylation in cardiomyocytes and mCPCs was analyzed using Roche NimbleGen 2.1 M mouse genome CHARM 2.1M array and NimbleGen DNA Methylation CpG Islands Pus RefSeq Promoter array (3×720 K). Labeled DNA samples were hybridized to arrays according to NimbleGen Array User Guide, and arrays scanned using MS 200 Microarray Scanner (Roche NimbleGen) and features extracted by NimbleScan software.

### Validation of gene expression by TaqMan RT-qPCR and TLDA at single-cell level

cDNA synthesis from individual cells of additional single cardiomyocytes and mCPCs was performed using the Cell-to-Ct protocol and pre-amplification option (Ambion). The cardiomyocyte-specific gene (*Myh6*) and putative progenitor and stem cell genes (*c-kit* and *Sox2*) together with two endogenous controls (*Actb* and *Gapdh*) were validated by TaqMan RT-qPCR assays (Applied Biosystems) at single-cell level. Moreover, a panel of genes for stem cell pluripotency and differentiation included in TaqMan Low Density Array (TLDA, 96 genes including house-keeping genes such as GAPDH; Applied Biosystems) were profiled for a separated subset of single cells using our optimized streamline protocols including single-cell lysis, cDNA synthesis, and pre-amplification. Real-time qPCR was performed on a 7900HT Fast Real-Time PCR System (Applied Biosystems) and data was collected and analyzed using SDS 2.3 software suite. Ct values were normalized to endogenous controls, and comparative 2^−ΔΔCt^ method was used to evaluate the relative gene expression in mCPCs versus cardiomyocytes^6^. DataAssist 2.0 (Applied Biosystems) was used to analyze the expression changes. As for comparison, normalized intensity of probe(s) of specific genes was used to calculate gene expression fold changes detected by microarray.

### Microarray data analysis

#### NimbleGen Mouse CHARM 2.1M and 3×720 K Arrays

The CHARM package in R/Bioconductor was used to generate QC reports for NimbleGen Mouse CHARM 2.1M Array to identify globally differentially methylated regions of the genome for each biological sample^36^. The differentially methylated regions were defined by a criterion of unadjusted *P*-value less than 0.05 in either type of array. The regions (genes) were defined as differentially methylated if a CHARM peak was within 2,000 bases pairs upstream and/or 500 base pairs downstream of the transcription start site.

Partek Genomics Suite software (Version 6.6; Partek, Inc., St Louis, MO) was used to analyze the NimbleGen Mouse 3×720 K Array. The Loess normalization method was used to preprocess the raw intensity data and obtain M-values. The ANOVA method was used to identify differentially methylated regions by criterion of *P*-values less than 0.001 and |*MAT scores*|>2.

*Affymetrix Mouse 430 2.0*. The Partek Genomics Suite was used to assess the quality of the spike-in control probes used in the Affymetrix Mouse Genome 430 2.0 Array. The raw intensity data from each biological sample was preprocessed using Partek’s RMA method as a means to generate background-normalized gene expression data. Differential gene expression was analyzed using the ANOVA method in the Partek Genomics Suite. Significant differentially expressed genes were selected if they had fold-change values greater than 2 plus *P*≤0.05 with FDR value as ≤0.05.

## Data Records

We deposited the raw data sets including two different DNA methylome microarray data sets (NimbleGen Mouse CHARM 2.1M and 3×720 K Arrays) in pair format, and an Affymetrix GeneChip Mouse Genome 430 2.0 in CEL format (see Data Citation 1,Data Citation 2, and Data Citation 3). Each dataset contains 6 samples in two groups, i.e., mCPC versus control cardiomyocytes, with 3 biological replicates in each group. We provided qPCR and TLDA data with descriptions in figshare as well (see Data Citation 4).

## Technical Validation

### Quality Metrics for NimbleGen Mouse CHARM 2.1M and 3×720 K Arrays

**Figure 2:**
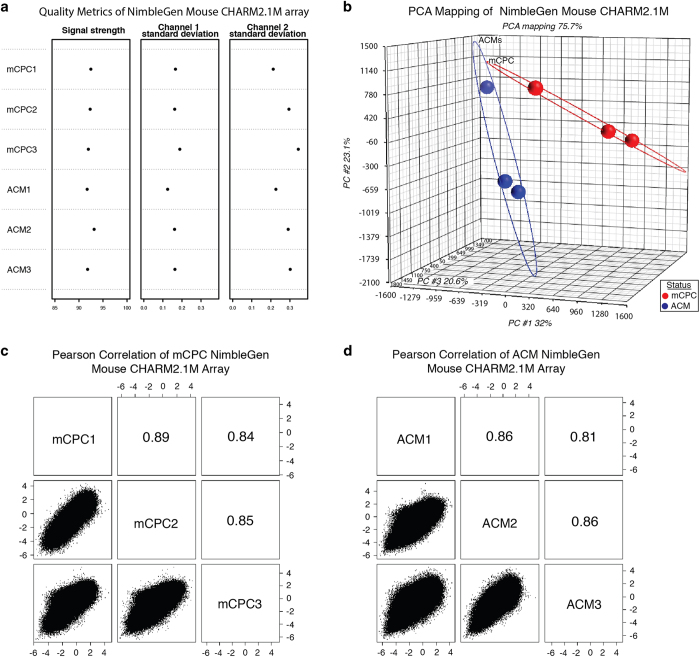
NimbleGen Mouse CHARM 2.1M Array quality metrics. (**a**) Summary plot with the control probe-sets and intensity data for all six samples. First column depicts average signal strength of the untreated channel signal probes among background probes. Second column depicts standard deviation of the untreated channel signal. Third column depicts standard deviation of the methyl-depleted signal. (**b**) Principal Component Analysis (PCA) of mCPCs compared to ACMs. (**c**) and (**d**) Pearson correlation of three biological replicates of whole population cells of ACMs and mCPCs, respectively.

**Figure 3:**
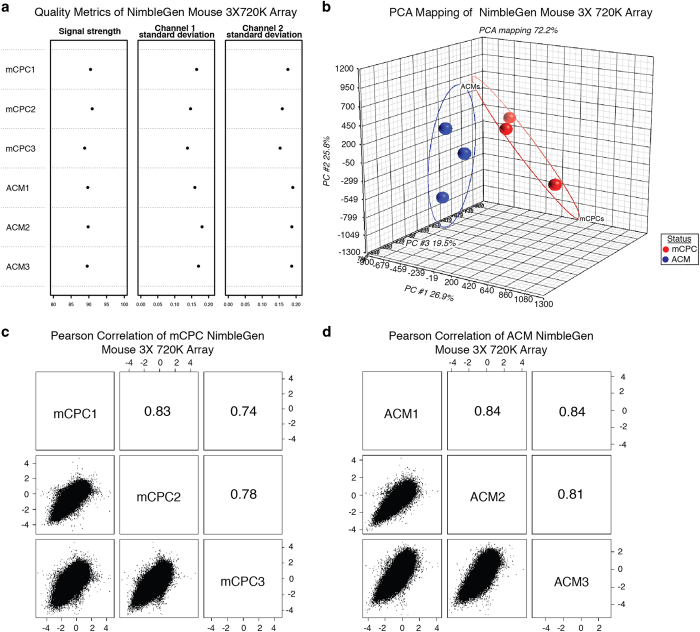
NimbleGen Mouse 3×720 K Array quality metrics. (**a**) Summary plot with control probe-sets and intensity data for all six samples. First column depicts average signal strength of the untreated channel signal probes among background probes. Second column depicts standard deviation of the untreated channel signal. Third column depicts standard deviation of the methyl-depleted signal. (**b**) Principal Component Analysis (PCA) of ACMs and mCPCs. (**c**) and (**d**) Pearson correlation of three biological replicates of whole population cells of ACMs and mCPCs, respectively.

QC metrics were generated using the CHARM R/Bioconductor package for the two types of NimbleGen tiling arrays which were used to analyze each biological sample’s methylome. Figures 2a and 3a show the three quality metrics that were calculated for the NimbleGen Mouse CHARM 2.1M and 3×720 K Arrays. The signal strength metric (column 1 in Figs 2a and 3a) represents the average percentile rank of untreated channel signal probes among the background probes, indicating the quality of hybridization and scanning. In ideal cases, a score of 100 means all signal probes rank above all background probes. In the NimbleGen Mouse CHARM 2.1M and 3×720 K Arrays, the signal strength scores range from 88.8 to 93.2, indicating acceptable hybridization and scanning for each sample. The signal standard deviation of untreated (column 2 in Figs 2a and 3a) and methyl-depleted (column 3 in Figs 2a and 3a) channels indicates the level of spatial artifacts in each array. In our arrays, most of the standard deviations are around 0.2 and the maximum standard deviation is only 0.34, which suggests that the arrays had limited spatial artifacts.

### Quality metrics for Affymetrix Mouse Genome 430 2.0 data

**Figure 4:**
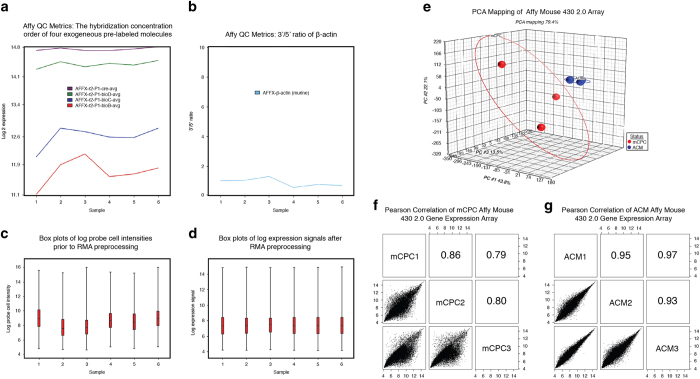
Affymetrix Mouse 430 2.0 Array quality metrics. (**a**) The hybridization concentration of four exogenous pre-labeled probes for all three single-cell biological replicates of ACMs and mCPCs samples. (**b**) RNA quality measuring 3′/5′ ratio of the beta-actin housekeeping gene within each of six samples. (**c**) Box plots of log probe expression cell intensities before RMA preprocessing (**d**) Box plots of log expression signals after RMA preprocessing (**e**) Principal Component Analysis (PCA) of ACMs and mCPCs. (**f**) and (**g**) Pearson correlation of three single-cell biological replicates of ACMs and mCPCs, respectively.

QC metrics were generated based on CEL files using Partek Genomics Suite, which allow us to summarize intensity values of the Affymetrix control probe-sets. A summary plot with the control probe-sets and intensity data for all six samples is depicted in Fig. 4. Figure 4a shows the hybridization concentration order of four exogenous, pre-labeled hybridization probes, which is consistent with the concentrations of the four spike-in probes (BioB<BioC<BioD<Cre). Figure 4b shows the 3′/5′ ratio of beta-actin with an acceptable ratio (<3) for all six samples, suggesting that the input RNA and cDNA derived from each single-cell sample used to generate the array data were of overall high quality. Microarray preprocessing is the most pivotal step in gene expression analyses. In our analysis, we applied the RMA method to preprocess the data, which includes background correction, normalization, and summarization. Figure 4c is the box plots of the probe intensities prior to the RMA preprocessing. Figure 4d shows the comparable range of probe sets intensities after RMA preprocessing.

### Biological replicates and reproducibility of single-cell transcriptome and population cell methylome data

The reproducibility of transcriptome and DNA methylome for three biological replicates within the same sample group can be used as another metric for assessing the quality of the data sets. A Principal Component Analysis (PCA) (Figs 2b, 3b and 4e) was performed to assess the quality of reproducibility of the biological replicates. Good separation between mCPC and control samples was observed after performing a PCA on single-cell transcriptome data and on the M-values of DNA methylome data using Partek Genomics Suite. Furthermore, we generated scatter plots and calculated the pair-wise Pearson correlation coefficient (R) between any two of the three biological replicates within the same group based on 2167126 features of NimbleGen Mouse CHARM 2.1M Array, 716168 features of NimbleGen Mouse 3×720 K Array, and 45101 genes of Affymetrix Mouse Genome 430 2.0 Array (Figs. 2c,d and 3c,d, Fig. 4f,g). The mean *r*-value and standard error (s.e.) were calculated per group, yielding 6 mean *r*-values and 6 s.e. values with a grand mean and s.e. of 0.847 and 0.029, respectively, indicating a high-level of measurement consistency and reproducibility among the biological replicates.

## Usage Notes

Briefly, we used Partek Genomics Suite and R/Bioconductor to perform data analysis in ‘Technical Validation’ section. In quality metrics of ‘Technical Validation’ section, we applied Partek Genomics Suite QC module to analyze Affymetrix Mouse 430 2.0 data. We used ‘readCharm’ and ‘qcReport’ function in CHARM package in R/Bioconductor to load data and perform QC metrics for two NimbleGen methylation tiling arrays. In biological replicates and reproducibility validation of ‘Technical Validation’ section, Partek Genomics Suite was used to perform 3D PCA for all three data sets. The ‘pairs’ function in graphics package in R was used to generate Pearson correlation figures for all three data sets.

## Additional Information

**How to cite this article:** Chen, X. *et al.* Single-cell transcriptome and epigenomic reprogramming of cardiomyocyte-derived cardiac progenitor cells. *Sci. Data* 3:160079 doi: 10.1038/sdata.2016.79 (2016).

## Supplementary Material
